# Supplementary material for: The individual determinants of morning dream recall
Source: Commun Psychol. 2025 Feb 18;3:25. doi: 10.1038/s44271-025-00191-z (PMC11836467; doi:10.1038/s44271-025-00191-z)
Supplement: Supplementary file 2 — Supplementary Materials [file 44271_2025_191_MOESM2_ESM.pdf]

SUPPLEMENTARY MATERIALS

The individual determinants of morning dream recall

Valentina Elce [1], Damiana Bergamo [1,2], Giorgia Bontempi [1], Bianca Pedreschi [1], Michele Bellesi [3], Giacomo Handjaras [1], Giulio Bernardi [1*]

[1] MoMiLab Research Unit, IMT School for Advanced Studies Lucca, 55100 Lucca, Italy
[2] University of Padova, Department of General Psychology, 35100 Padova, Italy
[3] School of Biosciences and Veterinary Medicine, University of Camerino, 62032 Camerino, Italy

*** Correspondence:**
Giulio Bernardi
IMT School for Advanced Studies Lucca
Piazza San Francesco, 19
55100 Lucca - Italy
email: giulio.bernardi@imtlucca.it

Supplementary Figure 1

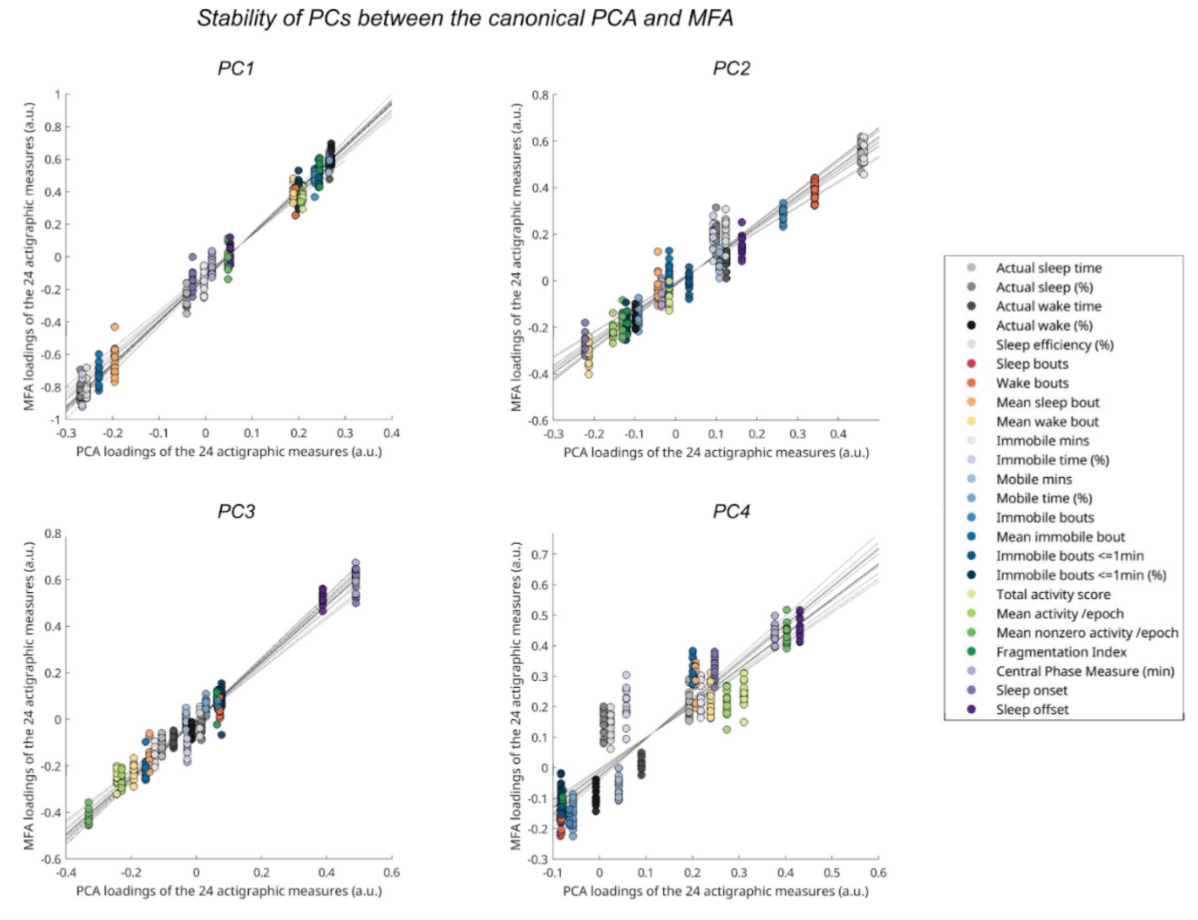

Supplementary Figure 1. Pearson correlations between the first four principal components (PCs) and MFA loadings. For each of the first 15 experimental nights, we generated a table with 200 rows (participants) and 24 columns (actigraphy measures), excluding ~1% of data from participants with extended experiment durations to minimize missing values. MFA was performed in R (FactoMineR library[1]) using default parameters (5 factors). Quantitative actigraphic variables were scaled to unit variance. Procrustes analysis was applied to align PCA and MFA loadings (allowing rotation and reflection) before measuring correlations. Each line in the plots represents the linear fit between PCA and MFA loadings for one night, with dots showing individual loading estimates for the 24 actigraphy variables across the first four PCs/factors.

Supplementary Figure 2

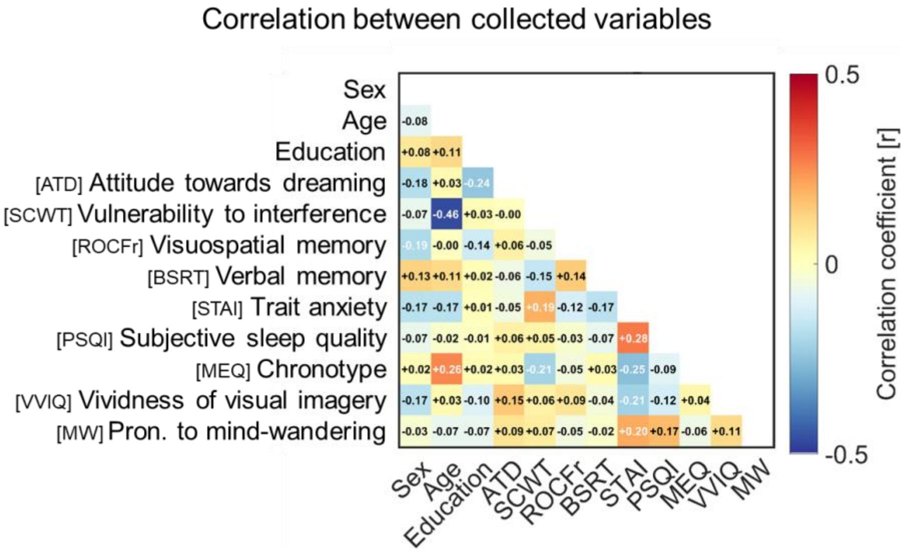

Supplementary Figure 2. Correlation (Spearman's correlation coefficient) between demographic, psychological and cognitive variables derived from questionnaires and tests. This plot is the same depicted in Fig. 1c, but here correlation coefficients are shown in each cell. White text indicates significant effects (q < 0.05, FDR correction).

Supplementary Figure 3

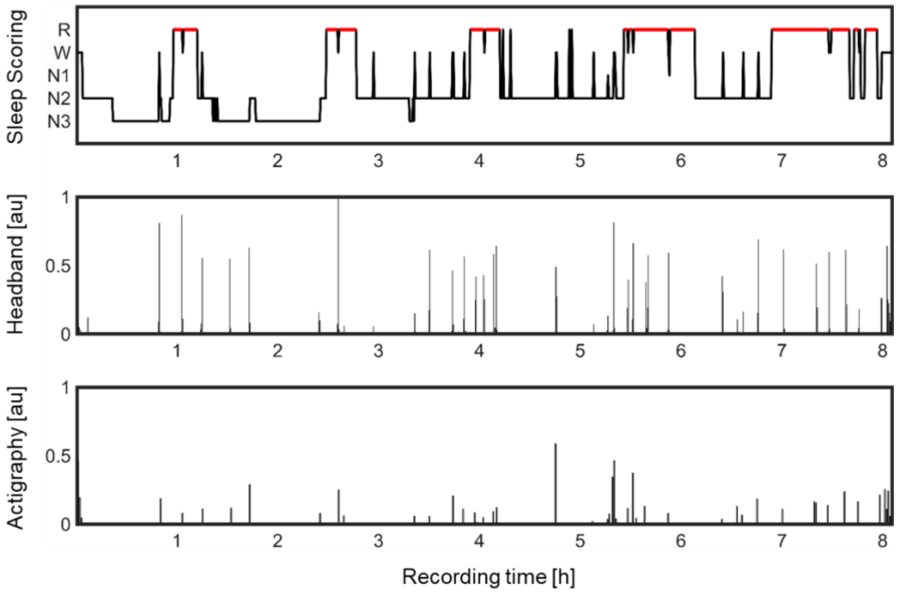

Supplementary Figure 3. Representative data from good quality data from one participant (DL005). The first row shows the sleep scoring obtained from the DREEM data. The second row shows the accelerometer data derived from the DREEM device. To facilitate comparisons, accelerometer time series values were normalized to a range of 0 (min) to 1 (max). The last row shows the aligned accelerometer data derived from the actigraphy.

Supplementary Figure 4

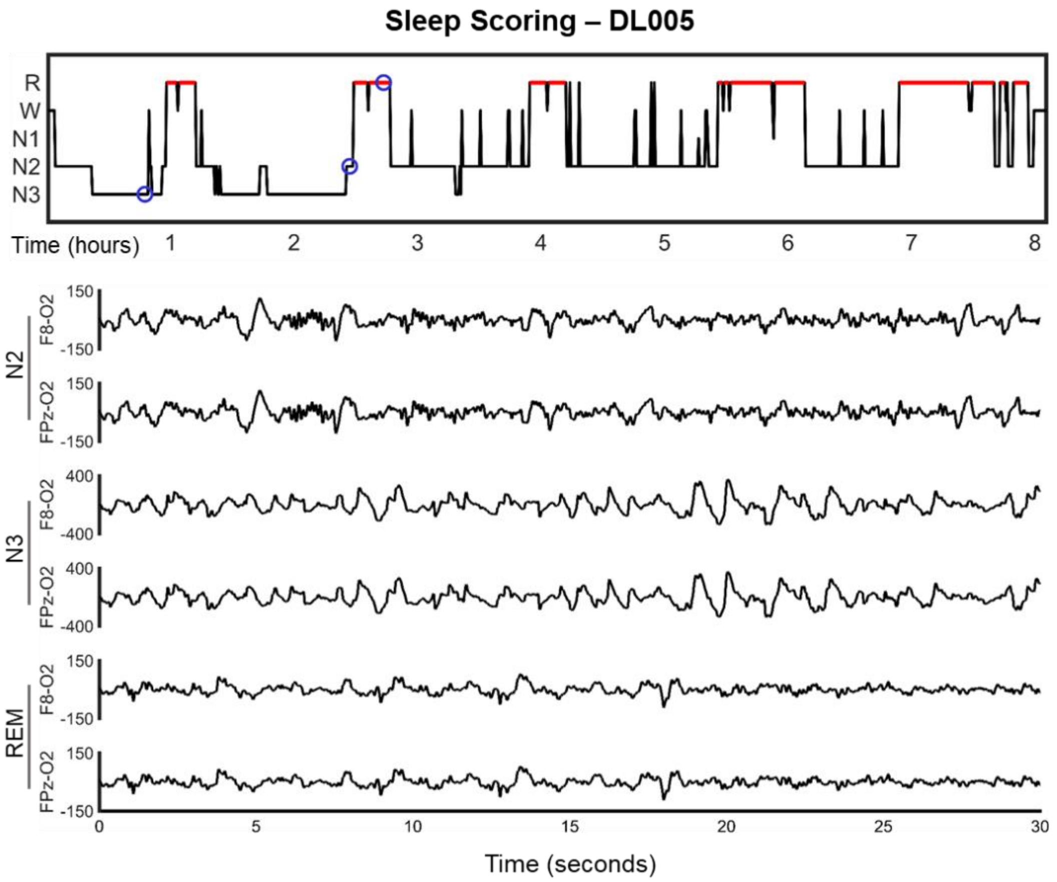

Supplementary Figure 4. Representative EEG traces (µV) from the same participant and night shown in Figure S2 (DL005). The top panel shows the sleep scoring obtained from the DREEM data where blue circles mark the 30 s epochs from which EEG traces were extracted.

Supplementary Figure 5

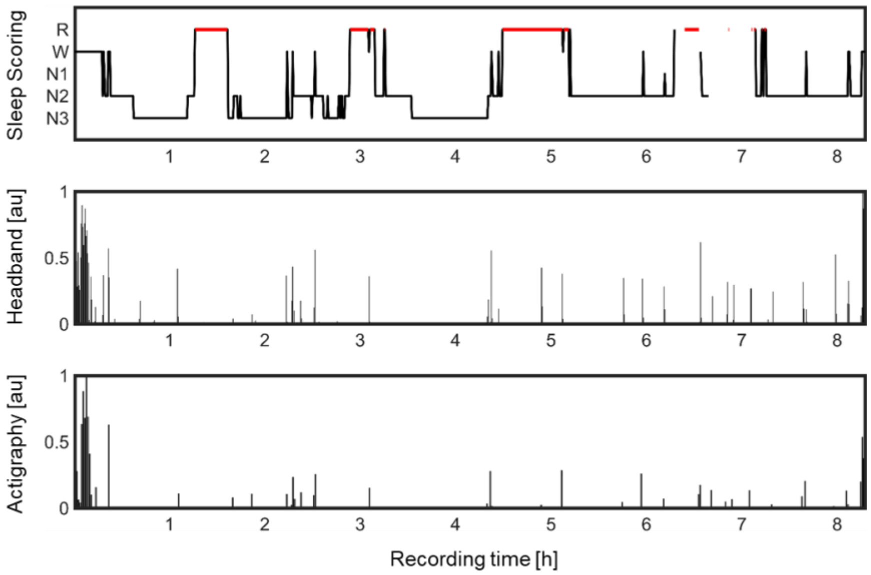

Supplementary Figure 5. Representative data from medium quality data from one participant (DL014). The first row shows the sleep scoring obtained from the DREEM data. The second row shows the accelerometer data derived from the DREEM device. To facilitate comparisons, accelerometer time series values were normalized to a range of 0 (min) to 1 (max). The last row shows the aligned accelerometer data derived from the actigraphy. Here some epochs in the 6th and 7th hour of recording were marked as unscorable.

Supplementary Figure 6

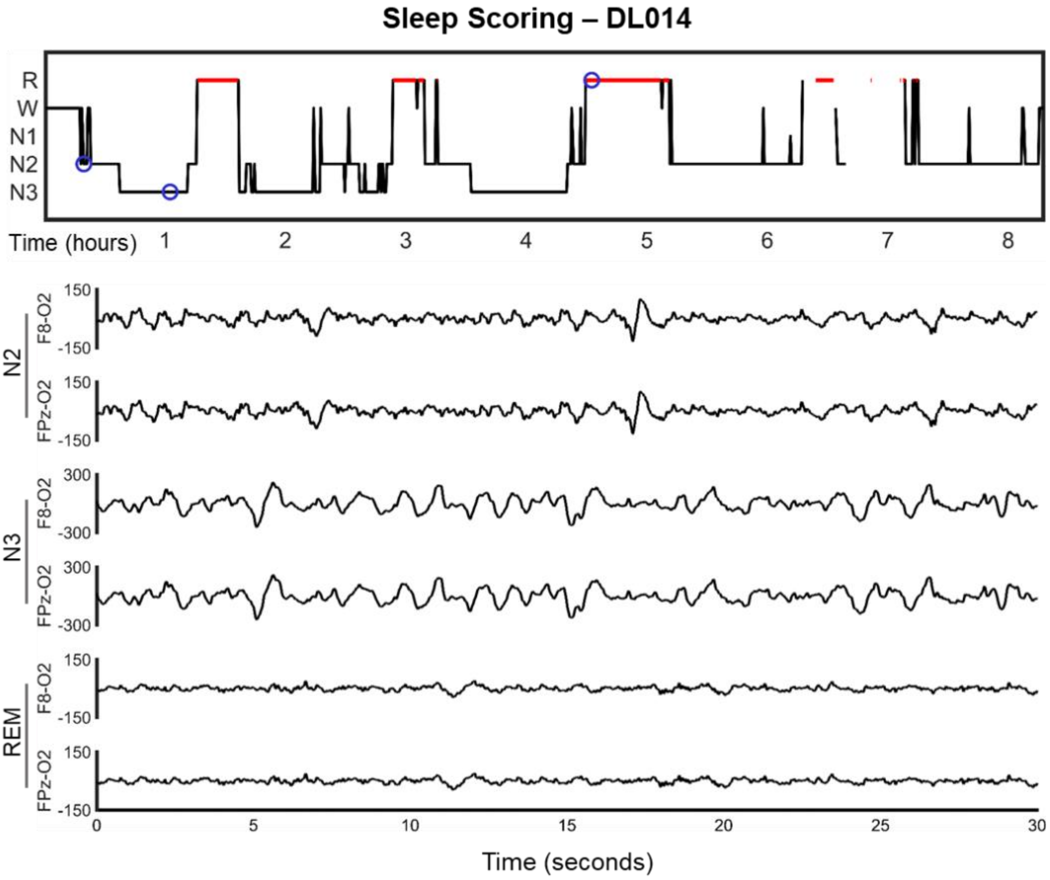

Supplementary Figure 6. Representative EEG traces (μV) from the same participant and night shown in Figure S4 (DL014). The top panel shows the sleep scoring obtained from the DREEM data where blue circles mark the 30 s epochs from which EEG traces were extracted.

Supplementary Figure 7

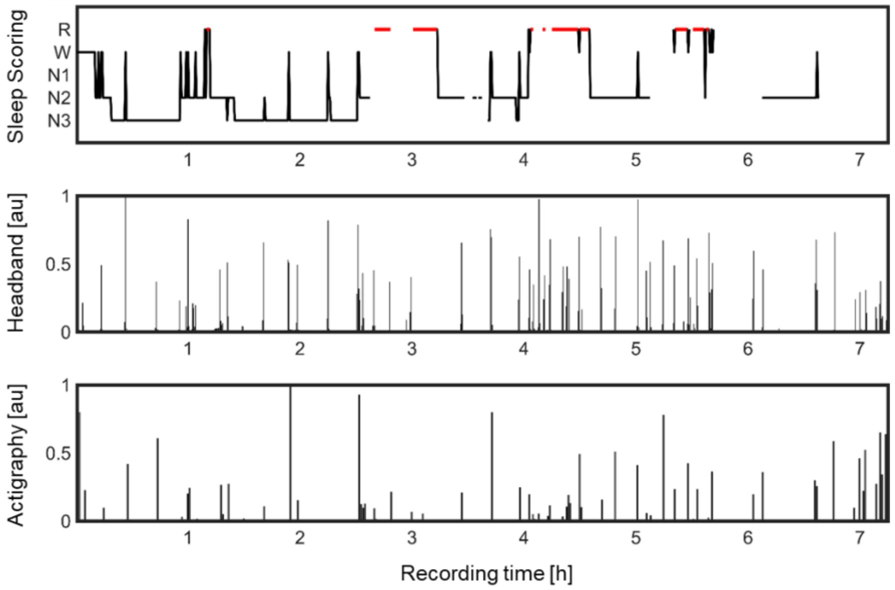

Supplementary Figure 7. Representative data from low quality data from one participant (DL006). The first row shows the sleep scoring obtained from the DREEM data. The second row shows the accelerometer data derived from the DREEM device. The last row shows the aligned accelerometer data derived from the actigraphy. To facilitate comparisons, accelerometer time series values were normalized to a range of 0 (min) to 1 (max). Here several epochs after the second hour of sleep were marked as unscorable (these epochs represented less than 25% of all sleep epochs), probably due to a high amount of body movements during the night. Cases like these were rare in our analyzed sample.

Supplementary Figure 8

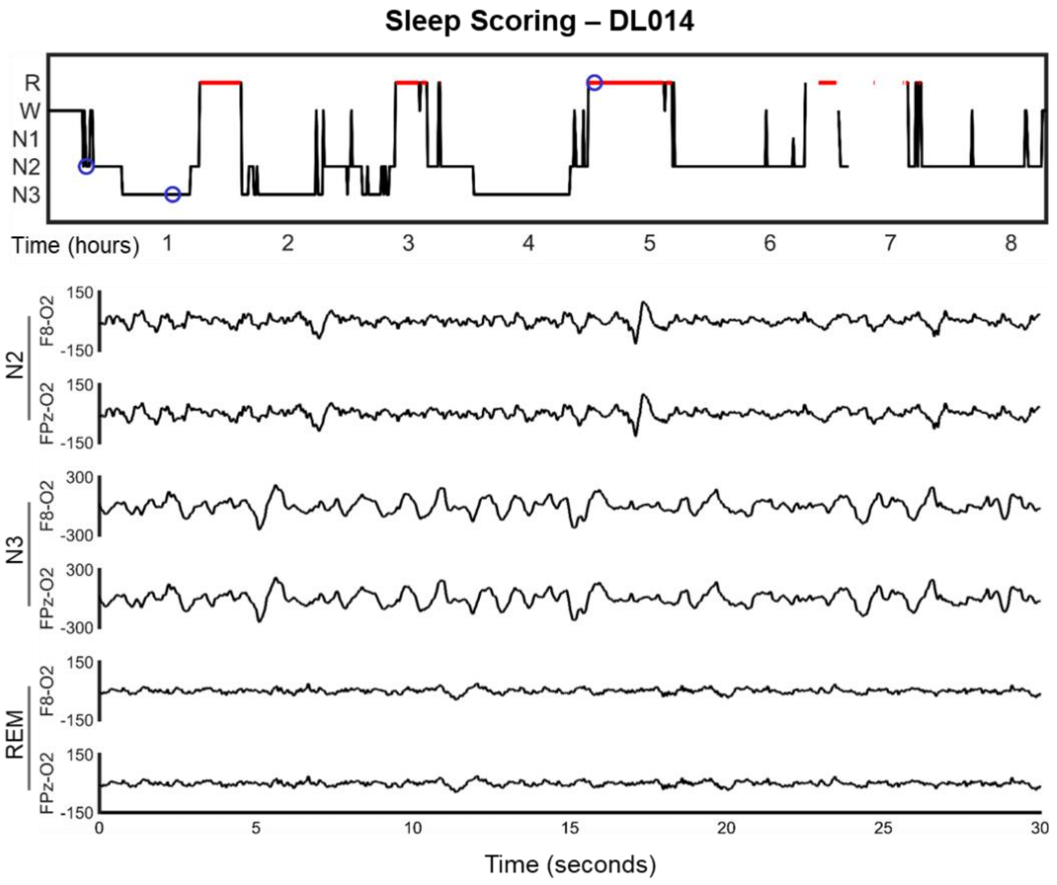

Supplementary Figure 8. Representative EEG traces (μV) from the same participant and night shown in Figure S6 (DL006). The top panel shows the sleep scoring obtained from the DREEM data where blue circles mark the 30 s epochs from which EEG traces were extracted.

Supplementary Figure 9

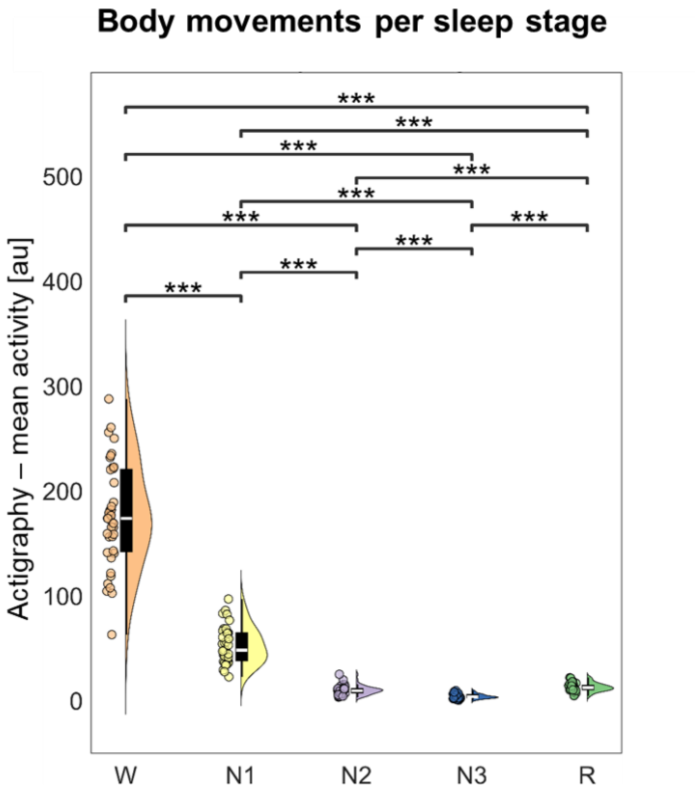

*Supplementary Figure 9. Mean accelerometer activity (as derived from actigraphic data) for each sleep stage defined according to the automated DREEM sleep scoring. The amount of activity differed significantly across all pairs of sleep stages, in line with previous research. * q < 0.05, ** q < 0.01, *** q < 0.001.*

Supplementary Table 1

Index	Stat.	W	N1	N2	N3	REM	Total
**Minutes [min]**	Mean	21.0	13.9	180.8	83.9	105.1	404.8
	SD	8.0	10.4	32.5	20.8	20.9	45.1
**Percentage [%]**	Mean	5.1	3.3	43.2	21.4	25.0	98.0
	SD	1.9	2.4	5.1	5.5	4.1	3.0

Supplementary Table 1. Sleep structure metrics computed for the 42 participants who wore the portable EEG device at night (480 nights in total).

References

1. Lê, S., Josse, J., & Husson, F. FactoMineR: an R package for multivariate analysis. *Journal of statistical software* **25**, 1-18 (2008).
