# Supplementary material for: The individual determinants of morning dream recall
Source: Commun Psychol. 2025 Feb 18;3:25. doi: 10.1038/s44271-025-00191-z (PMC11836467; doi:10.1038/s44271-025-00191-z)
Supplement: Supplementary file 3 — Reporting Summary [file 44271_2025_191_MOESM3_ESM.pdf]

nature portfolio

Corresponding author(s): Giulio Bernardi

Last updated by author(s): Oct 23, 2024

Reporting Summary

Nature Portfolio wishes to improve the reproducibility of the work that we publish. This form provides structure for consistency and transparency in reporting. For further information on Nature Portfolio policies, see our Editorial Policies and the Editorial Policy Checklist.

Statistics

For all statistical analyses, confirm that the following items are present in the figure legend, table legend, main text, or Methods section.

n/a	Confirmed	
☐	☒	The exact sample size (*n*) for each experimental group/condition, given as a discrete number and unit of measurement
☐	☒	A statement on whether measurements were taken from distinct samples or whether the same sample was measured repeatedly
☐	☒	The statistical test(s) used AND whether they are one- or two-sided<br>*Only common tests should be described solely by name; describe more complex techniques in the Methods section.*
☐	☒	A description of all covariates tested
☐	☒	A description of any assumptions or corrections, such as tests of normality and adjustment for multiple comparisons
☐	☒	A full description of the statistical parameters including central tendency (e.g. means) or other basic estimates (e.g. regression coefficient) AND variation (e.g. standard deviation) or associated estimates of uncertainty (e.g. confidence intervals)
☐	☒	For null hypothesis testing, the test statistic (e.g. *F*, *t*, *r*) with confidence intervals, effect sizes, degrees of freedom and *P* value noted<br>*Give P values as exact values whenever suitable.*
☒	☐	For Bayesian analysis, information on the choice of priors and Markov chain Monte Carlo settings
☐	☒	For hierarchical and complex designs, identification of the appropriate level for tests and full reporting of outcomes
☐	☒	Estimates of effect sizes (e.g. Cohen's *d*, Pearson's *r*), indicating how they were calculated

Our web collection on statistics for biologists contains articles on many of the points above.

Software and code

Policy information about availability of computer code

Data collection	No software was used for data collection.
Data analysis	The actigraphic indices were computed using the MotionWare Software (version 1.3; CamNtech). The DREEM headband was used to collect sleep EEG data and perform sleep scoring. Custom code written in MATLAB 2021b (MathWorks Inc., Natick, MA, USA) was used for statistical analyses. The packages DataViz (https://github.com/povilaskarvelis/DataViz), Cbrewer2 (https://it.mathworks.com/matlabcentral/fileexchange/58350-cbrewer2), and Sigstar (https://it.mathworks.com/matlabcentral/fileexchange/39696-raacampbell-sigstar) were used for data visualization. The Measures of Effect Size (MES) Toolbox was used to compute effect sizes (https://github.com/hhentschke/measures-of-effect-size-toolbox).

For manuscripts utilizing custom algorithms or software that are central to the research but not yet described in published literature, software must be made available to editors and reviewers. We strongly encourage code deposition in a community repository (e.g. GitHub). See the Nature Portfolio guidelines for submitting code & software for further information.

Data

Policy information about availability of data

All manuscripts must include a data availability statement. This statement should provide the following information, where applicable:

- Accession codes, unique identifiers, or web links for publicly available datasets
- A description of any restrictions on data availability
- For clinical datasets or third party data, please ensure that the statement adheres to our policy

All data supporting the findings of this study and the custom code used for data analysis were deposited to OSF: https://osf.io/3qwsm

Research involving human participants, their data, or biological material

Policy information about studies with human participants or human data. See also policy information about sex, gender (identity/presentation), and sexual orientation and race, ethnicity and racism.

Reporting on sex and gender	Self-reported biological sex was collected upon recruitment of study participants. Information about gender was not collected. The sex variable was included either as a variable of interest or potential confounding factor in all the performed analyses. Of the 204 participants included in the main analyses, 113 were females (55%). Of the 42 participants who provided usable overnight EEG recordings, 24 were females (57%).
Reporting on race, ethnicity, or other socially relevant groupings	Participants were recruited from the general healthy adult population. Only Italian native language speakers were recruited to ensure optimal linguistic comparability across dream reports. Race and ethnicity were not assessed as we did not expect such variables to affect our results.
Population characteristics	Only healthy adults with age comprised between 18 and 70 years old were included in the study. Only individuals with regular sleep/wake patterns, six to eight hours of sleep per night, and no diagnosis of sleep-related problems or of any other pathological condition that might have compromised their sleep were recruited in the study. Moreover, we excluded volunteers who were taking medications that could have affected sleep patterns at the time of the study and individuals who had a recent (last 6 months) history of alcohol and drug abuse. Finally, women who were pregnant, were planning a pregnancy, or were breastfeeding at the time of the study were also excluded. Age and sex were included either as a variables of interest or potential confounding factors in all the performed analyses.
Recruitment	Volunteers were recruited through word of mouth and advertisements. Potential differences in the individual interest in dreams was accounted for by evaluating the attitude towards dreaming of all the included study participants and including this factor in the performed analyses.
Ethics oversight	Local Joint Ethical Committee for Research of Scuola Normale Superiore and Scuola Superiore Sant'Anna (#11/2020)

Note that full information on the approval of the study protocol must also be provided in the manuscript.

Field-specific reporting

Please select the one below that is the best fit for your research. If you are not sure, read the appropriate sections before making your selection.

☐ Life sciences ☒ Behavioural & social sciences ☐ Ecological, evolutionary & environmental sciences

For a reference copy of the document with all sections, see nature.com/documents/nr-reporting-summary-flat.pdf

Behavioural & social sciences study design

All studies must disclose on these points even when the disclosure is negative.

Study description	Quantitative, prospective, observational study
Research sample	Healthy adult Italian speakers were sampled from the general population (18-70 years old, ~50% females). We recruited a total of 217 volunteers (116 females). Of these, ten failed to comply with the experimental protocol, and three provided less than seven valid morning reports leading to a final sample of 204 participants (mean age 35.1 ± 94 12.5 y; 113 females, 55.4%) and 2900 reports (14.22 ± 1.44 reports per subject). A subgroup of 50 volunteers (27 females; age 29.7 ± 5.2 y, range 22-44 y) also had their sleep-related brain activity recorded through a portable EEG system. However, 8 participants interrupted EEG data collection due to discomfort while sleeping, leading to a usable sample of 42 participants (24 females; age 30.0 ± 5.2 y, range 22-44 y; 480 nights in total). No a-priori sample size calculation was performed: all participants and data point collected over a period of four years (see below) were used in the analyses. The achieved sample size is similar to those of the largest previous prospective studies on dream recall. However, to our knowledge, it is the largest reached by a multimodal investigation combining behavioral, cognitive, and electrophysiological measures.
Sampling strategy	A convenience sampling procedure was applied for the participants' recruitment. Data collection took place over four years, from March 2020 to March 2024.

Data collection	Data collection took place through computerized questionnaires, pen and paper tests, portable voice recorders, actigraphy, and a portable EEG system (DREEM device). No blinding procedures were necessary for the adopted study design. The collection of verbal reports was performed autonomously by participants upon spontaneous morning awakening.
Timing	Data collection took place over four years, from March 2020 to March 2024.
Data exclusions	We did not include in our study delayed dream reports, that are cases in which participants did not recall any experiences in the morning but spontaneously provided later in the day an additional report in which they stated to have recalled a dream experience. Moreover, we excluded actigraphic recordings collected in 39 nights across 28 participants due to missing or unreliable data. Finally, actigraphic data (all nights) was lost in four participants due to technical issues (4 females, age 22-30 y). One experimenter manually inspected the DREEM headband sleep scoring output and eliminated nights containing obvious issues related to possible device removal or malfunction. Moreover, we excluded nights for which less than 5 hours of sleep were recorded, and nights in which more than 25% of all the epochs were marked as unscorable.
Non-participation	Of the 217 recruited volunteers, 13 were excluded from the main analyses as they withdrew from the study or failed to comply with the study requirements (i.e., they provided less than 7 morning reports). Exclusion criteria were defined prior to the commencement of data analysis. Of the 50 participants who accepted to wear the portable EEG system during their sleep, 8 were excluded as they prematurely interrupted data collection due to discomfort during sleep.
Randomization	Participants were not allocated into experimental groups.

Reporting for specific materials, systems and methods

We require information from authors about some types of materials, experimental systems and methods used in many studies. Here, indicate whether each material, system or method listed is relevant to your study. If you are not sure if a list item applies to your research, read the appropriate section before selecting a response.

Materials & experimental systems

n/a	Involved in the study
☒	☐ Antibodies
☒	☐ Eukaryotic cell lines
☒	☐ Palaeontology and archaeology
☒	☐ Animals and other organisms
☒	☐ Clinical data
☒	☐ Dual use research of concern
☒	☐ Plants

Methods

n/a	Involved in the study
☒	☐ ChIP-seq
☒	☐ Flow cytometry
☒	☐ MRI-based neuroimaging

Plants

Seed stocks	*Report on the source of all seed stocks or other plant material used. If applicable, state the seed stock centre and catalogue number. If plant specimens were collected from the field, describe the collection location, date and sampling procedures.*
Novel plant genotypes	*Describe the methods by which all novel plant genotypes were produced. This includes those generated by transgenic approaches, gene editing, chemical/radiation-based mutagenesis and hybridization. For transgenic lines, describe the transformation method, the number of independent lines analyzed and the generation upon which experiments were performed. For gene-edited lines, describe the editor used, the endogenous sequence targeted for editing, the targeting guide RNA sequence (if applicable) and how the editor was applied.*
Authentication	*Describe any authentication procedures for each seed stock used or novel genotype generated. Describe any experiments used to assess the effect of a mutation and, where applicable, how potential secondary effects (e.g. second site T-DNA insertions, mosiacism, off-target gene editing) were examined.*
